# Impact of age at marriage and migration on HIV and AIDS epidemics in Japan

**DOI:** 10.1186/1475-9276-8-23

**Published:** 2009-06-10

**Authors:** Nazrul Islam Mondal, Hiroshi Takaku, Yasushi Ohkusa

**Affiliations:** 1 Department of Life and Environmental Sciences and High Technology Research Center, Chiba Institute of Technology, Tsudanuma, Narashino, Chiba; Japan; 2 Infectious Diseases Surveillance Center, National Institute of Infectious Diseases, 123-1 Toyama, Shinjuku-ku, Tokyo, Japan

## Abstract

The causes of wide variation in the rates of HIV and AIDS epidemics among Japanese and non-Japanese nationals are not well understood. So, this paper examines the associations and assesses the potential roles of mean age at marriage, and migration in the HIV and AIDS epidemics in Japan. For the purpose, bivariate and multivariate regression analysis have been performed using epidemiological panel data to build up the relationships among overall HIV and AIDS prevalence, mean age at marriage, and migration. The same analyses have done for non-Japanese nationals living with HIV and AIDS separately. These indicators were significantly correlated with mean age at marriage, and migration. Multivariate linear regression analysis identified non-Japanese nationals' HIV and AIDS prevalence and mean age at marriage as the two most prominent factors linked with the national HIV and AIDS epidemics. The findings of this study supported the hypotheses that a high average age at marriage in the population leads to long period of premarital sex and the non-Japanese nationals' high prevalence facilitating the spread of the HIV and AIDS epidemics in Japan.

## Introduction

**Figure 1:**
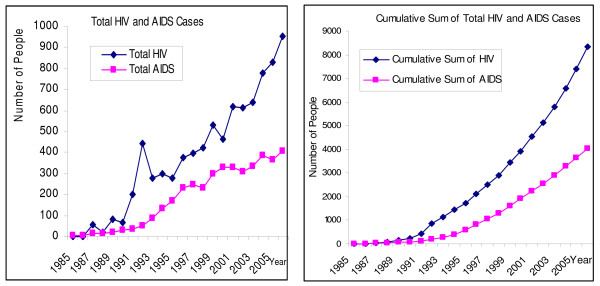
**Number of people living with HIV and AIDS Cases and their Cumulative Sum in Japan up to 2006 **[4].

**Figure 2:**
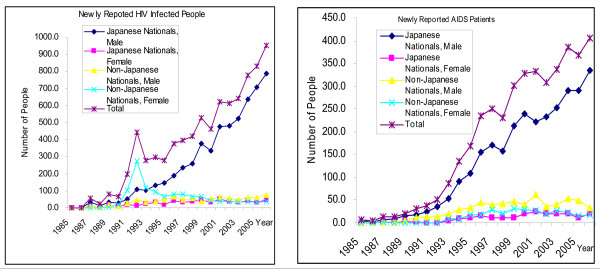
**Trends in the number of newly reported HIV infected people and AIDS Patients, 1985–2006 **[4].

**Figure 3:**
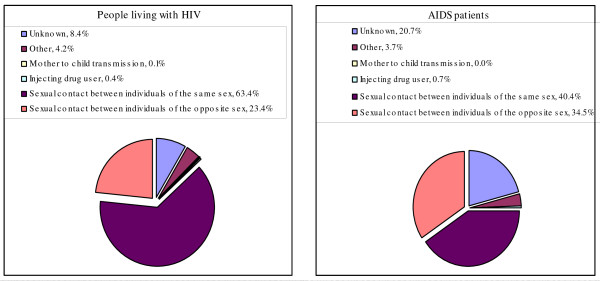
**Breakdown of infectious routes of PLHIV and AIDS patients reported in 2006 **[4].

Since the identification of the human immunodeficiency virus (HIV) in the early 1980s, much has been learned about how the virus is transmitted and how it attacks the body's immune system and causes the acquired immune deficiency syndrome (AIDS). The AIDS epidemic has grown on an unprecedented scale in three decades since it was first recognized, and is now considered a global crisis. In 2007, the total number of people living with HIV was 33.2 million, newly infected with HIV was 2.5 million, and AIDS deaths was 2.1 million [1]. Every day, over 6800 persons become infected with HIV and over 5700 persons die from AIDS, mostly because of inadequate access to HIV prevention and treatment services [1]. The HIV pandemic remains the most serious of infectious disease challenges to public health. Sub-Saharan Africa has experienced the most severe epidemic. In many developing countries, HIV prevalence was above 1%, but in none of the developed countries HIV has prevalence ever crossed the 1% mark [2]. The fastest growth of HIV among women occurs in East Asia, here women living with HIV jumped by 56% in 2 years [3]. It was 1985 that the AIDS Surveillance Committee, Ministry of Health, Labor and Welfare (MHLW) announced the first AIDS case in Japan. Though, they knew that many of hemophiliacs were infected with HIV even before 1985. The number of people living with HIV (PLHIV) and AIDS patients in Japan has continued to increase over time and upward trend [4]. While the total figure for reported cases is low compared with other advanced countries, the increase in newly reported AIDS cases is a phenomenon not seen in any other developed country. In those developed countries, the HIV infections have rapidly increased during the period between 1980s and the early 1990s, and a decline in the number of HIV/AIDS cases has been observed in the late 1990s. However, Japan did not experience such rapid increase but, instead, continued to increase slowly. The main route of transmission is sexual contacts, and enormously the sexual behavior of Japanese youth is drastically changing [5]. In recent years, HIV incidence rate has been rising dramatically, and the number of new HIV cases in Japan has been increasing year by year (Figures 1, 2). In 2004, the total number of new HIV/AIDS cases was over 1,000 and the cumulative reported number of HIV/AIDS cases was more than 10,000 [6]. The number of reported PLHIV has continued to increase since 1996, and the highest number of cases was reported in 2006, at 952 cases. The number consisted of 836 Japanese nationals and 116 foreign nationals. It is mentioned that 827 (86.8%) cases of infection were through sexual contact, of which 604 (63.4%) and 223 (23.4%) cases were between individuals of the same sex and of different sex respectively (Figure 3). The most significant increase in new HIV cases occurred among men who have sex with men (MSM), and 15 times more men than women reported a new HIV positive diagnosis in 2006 [4]. In addition, an increasing number of people ages 30 and older became HIV positive in 2006 compared with 2005, a nearly 10% increase in new HIV cases from 2005 to 2006, and a 6.3% increase for those who developed AIDS during the same time period [4]. A research group, funded by MHLW, predicts that the number of PLHIV will be approximately 30,000 by the end of 2006 in Japan. According to Hashimoto et. al. [7], there will be 50,000 HIV/AIDS cases by the year 2010 among Japanese nationals only. Several factors are believed to be conducive to large epidemics: high frequency of sexual intercourse outside marriage, multiple sexual partners, lack of condom use, lack of male circumcision, and infection with other sexually transmitted diseases (STD) [8]. The evidence for an important effect of genital ulcer disease and male circumcision practices is strong [9].

Currently, more than 100 million persons move voluntarily within or between nations each year and almost 40 million are either internally displaced or refugees outside their own countries. Many studies have revealed evidence of a potential association between human mobility and the epidemic [10,11], especially in the developing areas. The task of the study is to examine the hypothesis that the non-Japanese nationals' potential role in spreading the epidemics to the general population of Japan. The number of female migrant workers has been increasing, and many of them enter Japan and stay illegally and one avenue of illegal entry is through trafficking. Many of those women are forced into sex work as a means of survival, or have male partners who have had multiple sexual partners, resulting in exposure to high-risk sexual behavior [12]. The brothel-based commercial sex workers (CSW), and a huge number of freelance street-based CSW as well as illegal immigrant CSW in Japan who rarely visit STD clinics for routine screening purposed increased the risk of HIV transmission to the general population [13]. Kimoto et. al. [13] work on the CSW situation in Osaka has been well recognized, including the CSW's vulnerability and the situations relationships with the entry and spread of HIV. The present study also examines the hypothesis that late average age at marriage is another factor contributing to the spread of HIV because late marriage may lead to a long period of premarital sexual activity. Throughout the developing world marriage is the central social institution that regulates and sanctions sexual behavior. This suggests that age at marriage and sexual behavior before and after marriage could play a vital role in spreading HIV [14,15]. Bongaarts [16] has closely examined the relationship of age at marriage and HIV status. Ecological data indicate that the countries in southern Africa with very late age at marriage also have large epidemics. Thus, much has been known about what determines the spread as well as prevention of the HIV/AIDS epidemics. From best of our knowledge no study has been concentrated on the associations of mean age at marriage, and immigration with HIV and AIDS epidemics in Japan. So, it differs from other studies in several important aspects and it will use a broader range of explanatory variables based on more current and relevant data to offer a more comprehensive view of HIV and AIDS implications. By using best known statistical tools, it will be examined carefully how strongly mean age at marriage, and migration influence the sizes of HIV and AIDS epidemics in general and the scale of these diseases among non-Japanese nationals in particular. Hopefully, the study will focus on HIV and AIDS pandemics in Japan, and this can offer to the policy makers in socioeconomic options to combating both the diseases and its routes of infection.

### Study design and data

A dataset was created by compiling data from two different sources. The total number of HIV and AIDS and the number HIV and AIDS of non-Japanese nationals, and mean age at marriage were collected from MHLW [4] and the data on migration was collected from the Ministry of Justice [17] for the period 1985 to 2007. The ecological panel data has been used for bivariate and multivariate linear regression analysis. Keeping with the purpose of the study, two dependent variables were chosen for the multivariate linear regression analysis: (i) sizes of HIV and AIDS epidemics in a year as measured by total HIV and AIDS prevalence and (ii) share of non-Japanese nationals in the epidemics as measured by the proportion of population living with HIV and AIDS. Explanatory variables were (i) time, t measured in years; (ii) mean age at marriage as measured by years of the average age at first marriage of men, and (iii) migration as measured by the number of non-Japanese nationals living in Japan.

## Results

Bivariate and multivariate approaches have been applied in the analysis. Roles of time, mean age at marriage, and migration are examined here in turn.

### Bivariate Analysis

Correlation coefficients (*r*) were derived to examine direction, strength and significance of linear relationships between the variables included in the study (Table 1).

**Table 1:** Correlation between the variables that were examined

	Time	Total HIV Prevalence	Non-Japanese HIV Prevalence	Total AIDS Prevalence	Non-Japanese AIDS Prevalence	Age at Marriage	Migration
Time	1.00						

Total HIV Prevalence	.967**	1.00					

Non-Japanese HIV Prevalence	.341*	.436*	1.00				

Total AIDS Prevalence	.976**	.931**	.244	1.00			

Non-Japanese AIDS Prevalence	.880**	.797**	.316	.926**	1.00		

Age at Marriage	.976**	.964**	.211*	.955**	.795**	1.00	

Migration	.991**	.973**	.380*	.960**	.857**	.971**	1.00

** Significant at p < 0.01 level, * Significant at p < .05 level.

The significant similar relationships were found between time and the sizes of the HIV and AIDS epidemics for both the cases. The correlation was considerably lower for the size of the HIV epidemic among non-Japanese nationals (*r *= .341) than the overall HIV prevalence (*r *= .967). Again for the case of AIDS epidemic, the correlation was slightly lower for the size of AIDS epidemic among non-Japanese nationals (*r *= .880) than the overall AIDS prevalence (*r *= .976).

As expected, age at marriage is likely associated with the overall HIV prevalence (*r *= .964) and the non-Japanese nationals' share of the epidemic (*r *= .211) is very lower compared to the former. Again for the case of AIDS epidemic, the correlation was slightly lower for size of the AIDS epidemic among non-Japanese nationals (*r *= .795) than the overall AIDS prevalence (*r *= .955).

Migration is the most important issue in HIV and AIDS prevalence perspective. Correlation coefficients show that the higher migration, the greater HIV and AIDS epidemics. However, among the four explanatory variables considered, migration score appeared to have a significant, and the strongest, relationship with the overall HIV and AIDS prevalence (*r *= .973 and *r *= .960 respectively). But its correlation was significant and slightly lower with the proportion of HIV and AIDS prevalence who are non-Japanese nationals (*r *= .380 and *r *= .857 respectively).

### Multivariate Analysis

Four sets of multivariate linear regressions were conducted (Tables 2, 3). In the first two sets, the dependent variables were total HIV and non-Japanese nationals HIV prevalence, and in the second two sets were total AIDS prevalence and non-Japanese nationals AIDS prevalence.

**Table 2:** Multivariate linear regression models explaining the HIV epidemic

Explanatory Variables	Dependent Variables and Standardized Coefficients			
**Set 1**	**Non-Japanese Nationals HIV Prevalence**			

	Model 1	Model 2		Model 3

Age at Marriage	.211			-2800**

Migration		.380*		3.100**

Adjusted R^2^	-.003	.102		.544

**Set 2**	**Total HIV Prevalence**			

	Model 4	Model 5	Model 6	Model 7

Age at Marriage	.964**			1.150**

Migration		.973**		-.254*

Non-Japanese nationals HIV Prevalence			.435*	.290**

Adjusted R^2^	.926	.944	.150	.986

** Significant at p < 0.01 level, * Significant at p < 0.05 level.

**Table 3:** Multivariate linear regression models explaining the AIDS epidemic

Explanatory Variables	Dependent Variables and Standardized Coefficients			
**Set 3**	**Non-Japanese Nationals AIDS Prevalence**			

	Model 8	Model 9		Model 10

Age at Marriage	.795**			-.661

Migration		.857**		1.499**

Adjusted R^2^	.614	.721		.734

**Set 4**	**Total AIDS Prevalence**			

	Model 11	Model 12	Model 13	Model 14

Age at Marriage	.955**			.716**

Migration		.960**		.154

Non-Japanese Nationals AIDS Prevalence			.926**	.488**

Adjusted R^2^	.908	.918	.850	.986

** Significant at p < 0.01 level, * Significant at p < 0.05 level.

In the fist two sets of regression models, age at marriage and migration were included, and all these variables were significant predictors of HIV prevalence. For the case of non-Japanese nationals' HIV prevalence, the migration was stronger than that of age at marriage and better explained the model as indicated by the higher values of R^2 ^(Models 1–3). In the set 2 of regression models showed that migration and age at marriage were the more significant predictors compared to non-Japanese nationals' share in the total HIV epidemic (Models 4–7). When non-Japanese nationals' prevalence was included in the model, migration was no longer significant (Model 7), although it was significant independently (Model 5). Finally, age at marriage and non-Japanese nationals HIV prevalence are the most significant predictors for the total prevalence of HIV epidemic in Japan.

Similarly, in the second two sets of regression models, age at marriage and migration were included, and all these variables were significant predictors of AIDS prevalence. For the case of non-Japanese nationals' AIDS prevalence, the migration was stronger than that of age at marriage and better explained the models independently as indicated by higher values of R^2 ^(Models 8–9). But, when mean age at marriage and migration were included in the models, mean age at marriage was no longer significant (Model 10), although this was significant independently (Model 8). Thus, migration is the more significant for the non-Japanese nationals' AIDS prevalence. The set 4 of regression models showed that migration, and mean age at marriage were the two significant predictors compared with non-Japanese nationals share in the total AIDS epidemic (Models 4–7). When non-Japanese nationals' prevalence was included in the model, migration was no longer significant (Model 14), although this was significant independently (Model 12). As a final point, age at marriage and non-Japanese nationals' AIDS prevalence are the most significant predictors for the total prevalence of AIDS epidemic in Japan.

## Discussion

Good health is an important component of human wellbeing and the improvements in health and life expectancy are likely to contribute to greater economic growth and human resource development. HIV/AIDS affects not only the infected person, but also his or her family, community, and country. At the household level, people have loss of companionship and income. At the community and national levels, they experience loss of productivity because of absenteeism and deaths. The prevalence of HIV and AIDS are still low and little public attention is given to the epidemic as a serious issue confronting Japanese society. Experts point out that the epidemic may be spreading much more quickly than available figures indicate (Figures 1, 2, 3). Various underlying factors may be cited for the trend, the most prominent being changes in the sexual behavior of young people which fuel to the late marriage, greater migration across national borders, and delays in the identification of the infection.

Comparing other countries, Japan has three particular characteristics in HIV/AIDS situation: (i) a majority of reported HIV cases is infected through male-to-male sexual conducts, (ii) the higher prevalence rate among the non-Japanese nationals, and (iii) very few reported HIV cases is infected through intravenous drug use (IDU). The people considered being high risk and vulnerable to the epidemics are MSM, migrant workers, CSW and their clients. In the recent years, more than half of new HIV reported cases are infected through male-to-male sexual conducts. The main route of infection was sexual contact, in particular, MSM, which accounted for 63.4% of all PLHIV (Figure 3). Commonly cited statistics estimate the MSM population in Japan at approximately 1–2% of the total male population [18]. Many MSM reside in downtown Tokyo and Osaka, which are said to have the largest gay communities in Asia. As this number grows, there is an increasing need to improve the means of detection and the provision of swift treatment. The primary reason for this is that HIV infection is actually increasing among gay communities and MSM populations and thus gay communities in Japan have been exposed to the high risk of HIV infection. Heterosexual contact was the second most common mode of transmission (23.4%) and the rates of infection through IDU and mother to child transmission (MTCT) are both very low (<1%) (Figure 3). On the other hand, the key reason for few HIV infections through IDU is that the population of injecting drug users (IDUs) is comparatively small and they are isolated. Furthermore, in Japan, social stigma against drug users is very strong and drug controls is also very strict, thus the people with HIV may not report it, even if they were infected through drug injection. The proportions of foreign nationals who live in Japan are only 2%, but among all HIV/AIDS reported cases since 1985, the proportion of foreigners with HIV is 28.8% and the proportion of foreigners with AIDS is 24.1% and in 2004, among all HIV/AIDS reported cases, the proportion of foreigners with HIV was 13% and the proportion of foreigners with AIDS was 19.7% [4]. In terms of gender, Japanese men account for 89% of all cumulative HIV cases since 1985, as compared with Japanese women (11%). In contrast, foreign men account for 38% of all cumulative HIV cases, as compared with foreign women (62%). The possible reason for this difference is that the major route of HIV transmission among Japanese HIV/AIDS cases is male-to-male sexual conducts, where as foreigners are infected with HIV primary through sexual conducts between the opposite sexes. In terms of age group, those aged 30 – 39 years represented the highest number of cases of infection (41.0%), followed by those aged 15 – 29 years (29.6%).

The increase of PLHIV amongst Japanese males was most prominent; the number reported in 2006 (604 cases) greatly exceeded the previous year's figure (529 cases), and represented a record high. Also, the number of Japanese female PLHIV increased from 32 cases in 2005 to 49 cases in 2006. In terms of Japanese male PLHIV, the number of cases resulting from MSM (571 cases) had increased from the previous year (514 cases), representing the highest reported to date. Moreover, there were 173 cases of Japanese males infected through sexual contact with individuals of the opposite sex, up from 161 cases in the previous year. The number of Japanese female PLHIV infected through sexual contact with individuals of the opposite sex increased yearly until 1999, after which the numbers appeared to have stabilized. Figures (Figures 1, 2) reported in 2006, however, showed an increase in new cases, from 32 cases in 2005 to 49 cases in 2006. Looking at a gender breakdown by age groups of Japanese PLHIV infected through sexual contact with individuals of the opposite sex, females made up the majority in the 15–19 years and 20–24 years groups, which was in contrast with other age groups. The total number of AIDS patients reported in 2006 was 406, showing a continued increase from previous years, and representing the highest recorded level to date. Of this total, 355 (87.4%) were Japanese nationals, which reached the highest ever, and the number of foreign national AIDS patients decreased from 65 in 2005 to 51 in 2006. Out of AIDS patients reported in 2006, 74.9% of the patients were infected through sexual transmission, with 140 (34.5% of all cases) infected through sexual contact with individuals of the opposite sex and 164 (40.4% of all cases) with individuals of the same sex. Cases with unknown infection routes totaled 84 (20.7%). The assumed location of infection was within Japan for 315 cases (77.6%). The number of Japanese male AIDS patients was 335 (82.5%), increasing from the previous year (291). Out of these, 110 (32.8%) were infected through sexual contact with individuals of the opposite sex, 156 (46.6%) with individuals of the same sex, and 54 (16.1%) through unknown infection routes.

The trend of foreign nationals reported as PLHIV or affected by AIDS has flattened out. In 2006, there were 116 cases (12.2%) of foreign national PLHIV and 51 AIDS patients (12.6%) in Japan. These PLHIV were, in order of those nationalities most frequently reported, from Latin America, Southeast Asia, and the East Asia and Pacific Area excluding Japan. Among foreign AIDS patients, those from Southeast Asia were most frequently reported, followed by Sub-Saharan Africa, and the East Asia and Pacific Area excluding Japan. Looking at regional trends, Tokyo and the Kanto Koshinetu area (excluding Tokyo) remained areas with high levels of infection, representing 528 (55.2%) PLHIV and 211 (52.0%) AIDS patients in 2006. The number of PLHIV increased in Hokkaido, the Tohoku region, the Kanto Koshinetu area, Tokyo, and the Tokai and Kinki regions.

The average age at marriage has risen in most industrial countries, especially in Japan (28 year for female and 32 year for male). Even countries with less development, or who have come lately to industrialization show a rise in the average age at marriage for women. These statistics may reflect a greater feminist stance in most countries, with more women working and completing college. As well they reflect a trend away from marriage. Which results the considerably change the sexual behaviors among the youth and they participate in more sex with causal friends or multiple partners. In Japan, a network of youth having unprotected sex is rapidly expanding, leading to sharp increases in sexually transmitted infections (STI) and terminations of unplanned pregnancies [19]. As a result the abortion rate of teenagers has doubled and the number of Chlamydia and Gonorrhea cases among youth has also increased since 1995. Homosexual infection is expanding almost exponentially among Japanese males in age group of 20's and 30's, that is, before their marriages. HIV infection through homosexual route is increasing so acutely because of the changes occurring in the society of Japanese younger generations. Importantly, in the last several years, there are increasing incidents caused by "two-shot dials", "telephone clubs", "encounter web sites", and "legal drug" markets on webs, all related to the development of electronic webs [20]. Changing the sexual behaviors of Japanese youth is the key to reduce the expected HIV epidemic in Japan.

There has been a dramatic change in Japan's sex industry since the 1980's, especially in urban areas, where the traditional CSW is being marginalized by new type who offers manual stimulation, cunnilingus, and fellatia, but not vaginal intercourse. All types CSW are vulnerable population at the higher risk of HIV infection as well as transmission to their partners. The actual reported number of HIV/AIDS cases that involve sex workers is unknown. Japanese youth are actively involved in paid sex. Prostitution has been prohibited in Japan since the enforcement of the Anti-Prostitution Law in 1957. However, the sex industry has never disappeared and has continued to diversify since the 1980s as certain adult entertainment businesses were legitimized under a law regulating their establishments and services. More than 20,000 shops are registered throughout the country and it is said that many more operate illegally [21]. Among these businesses, it appears that the insistence on the use of condoms has not become a general practice and the risk of HIV infection is therefore high. Recently, informal sex work through the Internet is said to have dramatically increased among the younger generation, which is another serious cause for concern. However, the non-Japanese female sex workers are particularly vulnerable to infection as an ever widening segregation between them and native-born Japanese sex workers has led to tendency for the foreign nationals to engage in higher-risk practices.

Japan is simultaneously facing crises of exclusion and inclusion. By adopting more stringent laws to exclude foreign workers, it faces tremendous difficulties in coming to terms with its demographic future diminishing population and labor force. Yet because these laws invariably become less effective in stemming the entrance of foreign workers as both Japanese enterprises and recruitment networks find new ways to recruit them, it also perpetuates a system of illegal migration. While perhaps convenient to enterprises wishing to pay very low wages, this risks an equally problematic crisis of inclusion of foreign workers into everyday life accompanying the advent to Japan as a multicultural society. The non-Japanese nationals, most are believed to be migrant workers. Although a smaller portion, migrant workers, especially unregistered non-Japanese nationals without legal status, are similarly vulnerable and seriously at risk of HIV infection. A particularly high number of cases were reported in 1992, but since then the number has leveled off, meaning that this group cannot be used to explain the increasing number of HIV cases in Japan. In this period many women from Asian countries come to Japan as CSW and most of them were infected outside Japan [22]. There are two main factors that make migrant workers in Japan more vulnerable to HIV infection. One is the language barrier, which results in a lack of information on prevention and limited access to proper testing, treatment, and care. In many cases, they do not seek treatment until they are in the advanced stages of AIDS or need to be admitted to the emergency room. The second factor is treatment costs. Many migrant workers either do not have valid legal status or they possess visas that make them ineligible for public health insurance. The reluctance of such people to undergo testing or receive treatment because they cannot afford the costly medical bills, and of some hospitals to refuse such patients, has been a serious issue. Given this situation, many such non-Japanese have never been tested for HIV and tend not to visit medical institutions until they are in the advanced stages of AIDS or carried in he near death which is so much pathetic as human being. Everybody is at risk of contracting HIVAIDS regardless of nationality, race, ethnicity, gender, or age. One solution to the difficulties these populations find themselves in would be for the Infectious Disease Prevention Law to remove the condition of nationality and residency status from eligibility for health insurance.

In Japan, social awareness and public perception about HIV/AIDS is extremely low and few people voluntarily attempt to have HIV testing, except for a part of gay communities in the large cities. So, it is reasonably difficult to know the actual number of people with HIV, HIV infection rates, and the trends of HIV/AIDS at the early stages based only on the reported cases of HIV/AIDS. In reality, there is a big difference between actual number of HIV/AIDS and the trend, and the number of HIV/AIDS and the trend, which are represented by the Committee of AIDS Trends (CAT) and MHLW, etc. Thus, HIV/AIDS cases in Japan seem to continue increasing slowly but the increase rates are becoming faster and it seems to be just an iceberg. In Japan, HIV testing system is not strong and totally lacks counseling and the motivation for HIV testing is also weak. It is impossible to know the latest trend in the actual number of HIV/AIDS cases, based on the present surveillance systems, which depends on only reported HIV/AIDS cases. Using the present surveillance system, it is also impossible to know which population groups and communities are at the risk of increasing HIV infections. The government did not recognize the threat posed by the pandemic and only declared HIV/AIDS a national disaster. This is the major limitation to understand the current situation of HIV/AIDS in Japan. If the prevalence rates of HIV and AIDS epidemics rise tremendously, Japan must face the most critical situation, which is not expected.

## Conclusion

Among most developed countries, the number of newly reported HIV positive and AIDS patients has been decreasing, but it is increasing steadily in Japan. Based on the analyses of the present surveillance data, the HIV infections through sexual conducts tend to increase tremendously in the next few years. The major limitation to understand the current situation of HIV and AIDS is the present surveillance systems, which only depends on the reported HIV and AIDS cases. Added to this is a belief that only a low percentage of people testing to HIV in Japan actually report as required, suggesting that the number of HIV positive people in Japan is much higher than MHLW survey states. The continuous and simultaneous increase in both STD and HIV infection trigger the current situation and lead to a full-blown HIV epidemic, if no immediate action is taken. However, Japan is now in the low level of HIV and AIDS epidemics, it is considered as shifting to "concentrated epidemics". The study results suggest that the urgent need for introducing prevention measure for HIV and STD among youth which are currently appropriate for the target population. The findings support the hypothesis that a high average age at marriage in a population contributes to the spread of HIV because a higher age at marriage is strongly associated with a longer period of premarital exposure to the risk of infection. So, it is burning need to find out why the youth are disheartened to get marriage in proper time. Japan's anti-prostitution law needs to amend in the context of HIV/STD control because strict police security drives the problem underground and discourages street girls, especially those living in the country illegally, from seeking proper health services for HIV/STD. Consequently, HIV infection is rising enormously among non-Japanese females. Group-based interventions, better access to health care and a comprehensive public approach should be applied to the non-Japanese nationals. In fine, an AIDS center for prevention should be established to acquire knowledge and expertise and to demolish the epidemics.

## Competing interests

The authors declare that they have no competing interests.

## Authors' contributions

NIM performed statistical analysis and drafted the manuscript. YO developed the analytical approach and econometric methods. HT contributed to the drafting of the manuscript. All authors read and approved the final manuscript
